# Culture and self-construal in the age of globalization: an empirical inquiry based on multiple approaches

**DOI:** 10.3389/fpsyg.2024.1353898

**Published:** 2024-03-19

**Authors:** Kang Li, Fengyan Wang, Zhongling Pi

**Affiliations:** ^1^School of Education Science, Zhoukou Normal University, Zhoukou, China,; ^2^Institute of Moral Education, Nanjing Normal University, Nanjing, China,; ^3^Key Laboratory of Modern Teaching Technology (Ministry of Education), Shaanxi Normal University, Xi’an, China

**Keywords:** self-construal, polyculturalism, polycultural self-construal, cultural icon, individualism, relationism

## Abstract

**Introduction:**

There are three main types of culture in human society, namely, individual-oriented, relationship-oriented and social-oriented cultures. In history, there are two main positions on the relationship between culture and self-construal: the cultural determinist position and the interaction position. After analyzing literature critically, we propose that the interaction position is more persuasive than the cultural determinist position. A self-construal model was constructed from an interactionist and polycultural perspective, pointing out the relationship between three cultures and self-construal. We argue that individuals interacting with cultures in the context of globalization can develop a more integrated self-construal. The present study proposes the existence of polycultural self-construal, and aimed to explore how self-construal factors relate to cultures.

**Methods:**

Three approaches—psychological tests, priming with cultural icons and content analysis—were used to explore mechanisms between cultures (individual-oriented, relationship-oriented, and social-oriented cultures) and self-construal. In Study 1, we recruited 460 undergraduate students as participants through campus advertising to complete three psychological tests, namely, the Cultural Identity Scale (CIS), the Marlowe-Crowne Social Approval Scale (MC-SDS), and the Polycultural Self-construal Scale (PSCS). In Study 2, we created icon materials that could prime the three cultures. The experimental process was divided into two stages: priming and measurement. First, 165 participants were presented with icon materials on the computer screen to activate the corresponding culture, and then they were asked to complete the PSCS. In Study 3, the experimental procedures were followed as for Study 2. Then the Ten Statements Test (TST) was used. Each of the 178 participants gave 10 different responses to the question of “Who am I?.” Each participant’s “I am …” narratives were qualitatively processed using content analysis.

**Results:**

The individual-oriented culture mainly affects the individuality and equality factor of self-construal. The relationship-oriented culture mainly impacts the relationality factor of self-construal, while the social-oriented culture mainly affects the collectivity and equality factors of self-construal. There were no significant differences in the effects of the three cultures on the autonomy factor of self-construal. The multi-components of the polycultural self-construal are difficult to interpret based on one culture type. All three cultures have specific and shared effects on human self-construal.

## 1 Introduction

Culture shapes the human mind and self-expression. In the Middle Ages, there were traditions of male bearding both in China and the West, which were often reflected in portrait paintings or biographical descriptions before modern times. Chinese men wore beards associated with Confucian culture. The Book of Filial Piety clearly records “My skin and hair are given by my parents and cannot get any scratches. This is the beginning of filial conducts.” In the West, beards may have their origins in Hebrew cultural traditions. The Book of Leviticus in the Holy Bible makes this provision clear: “Thou shalt not mar the corners of thy beard.” However, cultural changes and a new emphasis on the rights of youth have influenced society, making contemporary male beards rare in China and many other countries. People in different parts of the world do not only grow beards for personal reasons, but to express their own cultural connotations. Culture and human self-construal are closely interlinked.

The term ‘self-construal’ originated from Markus and Kitayama (1991) who asserted that the self is the process and product of culture. They defined it as how individuals see the self in relation to others, and identified two types of self-construal: independent and interdependent. Independent self-construal refers to the self as a special entity that is detached from the environment with independent and autonomous characteristics, and related to Western individualistic culture. Interdependent self-construal refers to the self as a relational being connecting with others, society, and through shared backgrounds, and related to Asian collectivistic culture (Markus and Kitayama, 2010). The self, which is considered to be universal, has taken on diverse and rich cultural connotations, and analyzing the relationship between culture and self has become an important topic (Kagitçibasi, 1996; Hong and Mallorie, 2004; Krahnke et al., 2012; Park et al., 2016).

In history, there are two main positions on the relationship between culture and self: the cultural determinist position and the interaction position. The cultural determinism position, closely related to cultural anthropology and cross-cultural psychology, has been extremely influential. For example, Linton (1947, p. 98), a leading exponent of the ‘culture and personality’ school, wrote: “Culture must be considered the dominant factor in establishing the basic personality types for various societies and also in establishing the series of status personalities which are characteristic for each society”. Heine (2001) also argued that the self is a product of culture, and Kitayama et al. (2007) considered the self as a cultural mode of being. Cross et al. advocated that different self-construals reflect different cultural models of selfhood, and that individuals’ views of themselves are generated within a cultural framework influenced by values, concepts, structures, and conventions (Cross and Madson, 1997; Cross et al., 2011).

Under the cultural determinist position, culture is often understood as an external, collectively shared element, which may refer to a social structural presence or to a symbolic system with a structural nature. This view is similar to that of the American sociologist Parsons (1951), who argued that the cultural system is an important part of the social system and asserted that culture has three core elements: (1) culture is passed on to future generations as a heritage or social tradition with boundaries; (2) culture needs to be learned; it cannot express itself automatically, as in the case of the genetic structure of a human being; and (3) culture is shared by members of a society. From this, we can see that in the cultural determinist position, the influence of culture on the self is from the outside in, and the self shows the passivity of being surrounded and dominated by a certain culture.

Such an understanding of the relationship between culture and the individual tends to treat culture as a closed entity system, and to emphasize its own coherence and harmony. This has led us to mistakenly believe that East Asian culture is an interconnected and interdependent cultural system, while Western culture is an autonomous and independent cultural system. In this position, the way people face new culture is by means of what cross-cultural psychologists refer to as acculturation, ignoring the individual’s agency and subjectivity. In essence, culture does not account for psychological differences; it is rather a contextual part of it; the psyche emerges as a totality in dialogue WITH culture rather than as a product of cultural differences (Tateo, 2018). Thus, the cultural determinist position has been strongly challenged.

The interaction position is more persuasive than the cultural determinist position. Swidler (1986) asserted that culture and mind are inextricably intertwined and interactively constructed, and that the human mind is shaped by culture, while, at the same time individuals’ mental activities shape culture. Theories with a social constructivist orientation, such as Habermas’s (1984) communicative action theory and Gergen’s (2011) relational being theory, describe interaction between individuals and social culture. Some proponents of the interaction position treat culture as a medium of symbols, information, or knowledge that can be manipulated by individuals. Valsiner (2014, 2017) systematically critiqued the cultural determinist position, asserting the mutual generativity of culture and the individual mind, arguing that culture should be viewed as the individual’s tools for life rather than causal constructs. An important term in Valsiner’s view is ‘externalized’, which highlights the subject’s ability to actively reconstruct information when using symbolic interactions, demonstrating new mental behaviors that affect others around them.

Cultural psychologist Gamsakhurdia’s semiotic theory of the self emphasizes the function of the subject in the relationship between culture and the self, deepening the understanding of cultural psychology (Gamsakhurdia, 2018, 2019, 2021). He argues that mainstream acculturation studies have been criticized for taking a mechanical and essentialist view of socio-cultural transformations, and has therefore proposed the term ‘proculturation’ to emphasize the constructive and subjective nature of human adaptations to novelties. Proculturation develops when a person is confronted with any kind of novelties. It is a continuous process. Each proculturative experience inevitably imprints on self, as any encounter with new ideas is subjectively interpreted and becomes part of the cognitive and emotional experience. So, culture, as well as self, can never be self-sufficient and isolated as it is substantially formed and reconstructed through dialog.

In a contemporary globalized society, Morris et al. (2015) adopted a polycultural perspective, arguing that culture is a loose knowledge network and an open system, and that cultures are not categorical but rather partial and plural. Going back to the origins, Kelley (1999) and Prashad (2003) coined the concept of polyculturalism which means that human cultures are interrelated and mutually influential in the process of change and development. As Kelley (1999) stated, “All of us, and I mean ALL of us, are the inheritors of European, African, Native American, and even Asian pasts, even if we cannot exactly trace our blood lines to all of these continents” (p. 81). Its core assumptions relate to how cultures have interacted with each other throughout history through various forms of cultural contact (Bernardo and Presbitero, 2017; Cheon, 2019). The polycultural perspective highlights the interaction between individuals and cultures. In the face of diverse cultures resources, individuals have the ability to actively explore, select and integrate in order to realize their own life goals. Where once research in polycultural psychology focused on cross-cultural immigrant groups or individuals, contemporary psychologists advocate that such research can be extended to all groups or individuals involved in the globalization movement (Chiu and Kwan, 2016). From the polycultural perspective individual human beings do not need the direct lived experience of other cultures, but are influenced by exposure and interaction with other cultural symbols, resulting in a culturally integrated mindset (Li et al., 2020).

Polycultural psychology is more in line with phenomenologically methodologically oriented cultural psychology regarding the relationship between culture and self, but differs in that the former offers a number of empirical research methods. Based on this, we construct a polycultural theory that attempts to account for the relationship between self-construal and three common cultural types, and explore it empirically. The cultural traditions of Eastern and Western societies, despite their complexity, basically revolve around three levels of being: individuality, relationships, and society. The individual level involves the survival and development of oneself, the relationship level is the survival and development of one’s own small relational group, and the social level involves one’s nation, country, human beings, and even the earth’s ecosystem. Culture was created by humanity in response to the tension between the three levels. Thus, although there are many diverse human cultures, culture is always biased toward one of the three levels—toward individual-oriented culture, such as modern European and US culture, or a relationship-oriented culture, such as traditional Chinese Confucian culture, or a social-oriented culture, such as the national culture of some East Asian countries. For Western civilization, there were cultural changes with different orientations. Early Western civilization was very much focused on social-oriented city-state culture and family-oriented relational culture, but from around the 16th–18th centuries, Western civilization began to focus on individual-oriented culture (Baumeister, 1987). As for the Chinese civilization in East Asia, three kinds of orientation cultures were popular during the Eastern Zhou period, which were Yang-Zhu culture representing the individual orientation, Confucian culture representing the relational orientation, and Mohist culture representing the social orientation. In the change of Chinese cultural history, Confucian culture became the dominant culture in Chinese society after Emperor Wu of Han Dynasty, as an important factor to promote the formation of Chinese interdependent self-construal (Wang, 2019).

In the era of globalization driven by information technology, individuals are increasingly exposed to multicultural knowledge and experiences, and as a result, multicultural coexistence has become a common phenomenon in people’s lives. The Chinese society in which the author lives is rapidly integrating into the globalization process and inevitably affected by multiple cultures. Modern China is, thus, a typical polycultural society.

**Figure 1:**
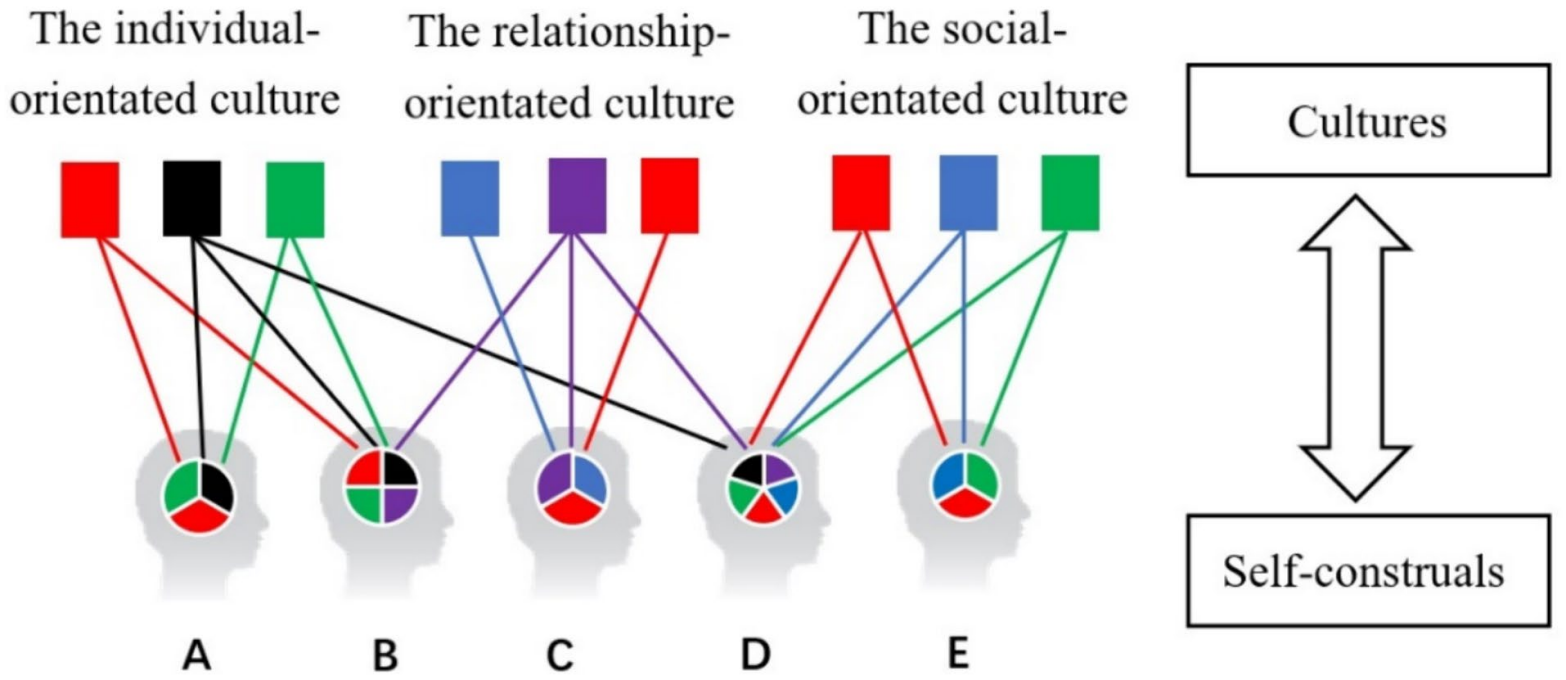
The polycultural self-construals model.

A self-construal model was constructed from an interactionist and polycultural perspective, as shown in Figure 1. This model indicates the relationship between cultures and self-construals, along with multiple types of self-construals, such as A, B, C, D and E, where B and D denote polycultural selves.

The present study proposes polycultural self-construals, in which the mechanisms linking self-construals to the three differently oriented cultures are yet to be scientifically proven. Two approaches were explored. First, cultural identity measurement was considered. Schwartz et al. (2006) argued that cultural identity can reflect the self-concept or self-construal. Individuals vary in their degree of multicultural self-integration, reflected in identification with multiple cultures (Mamat and Wu, 2017). Measuring an individual’s identification with the three differently oriented cultures and the polycultural self-construals can reveal the mechanisms by which the three cultures relate to specific components of self-construal.

The second approach involved cultural priming techniques. The cultural priming paradigm is a way to study the mechanisms underpinning culture and self-construal. Cultural identity research mainly involves self-assessment tests that explore the relationship between culture and self-construal at a conscious level, while cultural priming has an implicit effect that can extend beyond the level of consciousness. Hong et al. (2000) proposed a dynamic constructivist approach to culture, leading the trend of using priming experimental paradigms in cultural psychology. Cultural icons are images created or selected for their power to evoke a particular frame of mind in observers. The potency and distinctiveness of icons make them ideal candidates for primes that spread activation in a network of cultural constructs. The activation of culture is related to its availability, accessibility, and applicability. Activating varying knowledge networks results in different self-behavioral responses (Hong and Mallorie, 2004; Hong, 2009). Cultural icon priming has been widely used in recent years, especially in studies conducted with Chinese participants (Liu et al., 2017; Hu et al., 2018). These studies suggest that the cultural icon priming method is preferable for the exploration of mechanisms between culture and self-construal. Overall, cultural identity measurement emphasizes the role of the subject, while cultural priming emphasizes the role of the cultural object. Combining the two approaches may comprehensively illustrate mechanisms of culture and self-construal. Thus, the following three studies were undertaken using both approaches.

## 2 Study 1: measurement of the relationship between cultural identity and self-construal

### 2.1 Objective

To analyze the relationship between cultural identity and the components of polycultural self-construal.

### 2.2 Methods

#### 2.2.1 Participants

The empirical study followed the standard requirements of the Biomedical Research Ethics Committee of Nanjing Normal University. Through campus advertising we recruited 460 undergraduate students as participants [mean age: 20.73 years (SD = 1.63); 218 (52.6%) females].

#### 2.2.2 Tools

The Cultural Identity Scale (CIS) was adapted from the Multigroup Ethnic Identity Measure—Revised (MEIM-R) by Phinney and Ong (2007). Based on Zhang’s (2018) approach, we adapted selected terms to ensure they were suitable for measuring cultural identity (e.g., replacing ‘ethnic group’ with Confucian culture, national culture, or individualistic culture). The scale has two dimensions: exploration and commitment. Cronbach’s α coefficients for the two dimensions were 0.76 and 0.78, and for the total scale 0.81. Participants were asked to indicate the degree to which they agreed or disagreed with statements on a 6-point Likert scale. The AMOS 26.0 module was adopted for Confirmatory Factor Analysis (CFA), and a comparative fit index (CFI) of 0.942 was reported for the Confucian CIS; the incremental fit index (IFI) was 0.943, and the root mean square error of approximation (RMSEA) was 0.065. The CFI of the Individualistic CIS was 0.956, IFI was 0.956, and RMSEA was 0.087. CFI of the National CIS was 0.966, IFI was 0.965, and RMSEA was 0.074. Cronbach’s α for the three cultural identity subscales ranged from 0.89 to 0.93.

The Marlowe-Crowne Social Approval Scale (MC-SDS) (Wang et al., 1999, pp. 387–389; Greenberg and Weiss, 2012) has 13 items with a Cronbach’s α of 0.74 and a retest correlation coefficient 1 month later of 0.88. Participants were asked to rate statements on a 4-point Likert scale with one dimension. In this study, the AMOS 26.0 module was adopted for CFA, revealing that CFI was 0.889, IFI 0.889, RMSEA 0.097, and Cronbach’s α 0.80.

The Polycultural Self-construal Scale (PSCS) (Li and Wang, 2023) has five dimensions, including individuality, relationality, collectivity, autonomy, and equality. Cronbach’s α for the self-construal subscales under the five dimensions ranged from 0.76 to 0.84. Participants were asked the degree to which they agreed or disagreed with statements on a 6-point Likert scale. Cronbach’s α for the subscales in this study ranged from 0.65 to 0.82.

#### 2.2.3 Procedures

The group test was conducted in two universities in Shaanxi Province, China, and those who completed the scale were paid 3 CNY. Following the sampling method of Talhelm et al. (2014), the sample data collected excluded participants from the three border areas of Xinjiang, Inner Mongolia, and Tibet, and minority participants from other areas. Valid data from 460 participants were entered into the SPSS 22.0 program.

### 2.3 Results

#### 2.3.1 Analysis of the relationship between three cultural identities

Common method bias has received considerable attention in psychological research. To manage that problem—while also using anonymous assessments, assessment tools that were relatively quick for participants to complete, and contained ‘attention checks’ for participants—we used the statistical control method of Harman’s one-way test on the PSCS, and found that the proportion of variance explained by the first principal component to the total variance was 25.53%. This suggests the common method bias was not large because the proportion did not exceed 50%.

The three cultural identity outcomes are shown in Table 1.

**Table 1:** The basic situation of the three cultural identities (*n* = 460).

	*M*	*SD*	*1*	*2*	*3*
1 Confucian cultural identity	21.82	7.20	1		
2 Individualistic cultural identity	17.14	5.31	0.18^**^	1	
3 National cultural identity	28.60	5.78	0.46^**^	0.06	1

^*^p < 0.05, ^**^p < 0.01.

Table 1 shows that national cultural identity scored the highest, followed by Confucian and individualistic cultural identity. The three cultural identities did not show an antagonistic relationship with each other; there was a significant moderate positive correlation between Confucian and national culture identity, a very significant low positive correlation between individualistic and Confucian culture identity, and no significant correlation between national and individualistic culture identity.

#### 2.3.2 Multilevel regression analysis of the relationship between cultural identity and self-construal

To balance the effects of gender and growing up environment on self-construals, and eliminate the effect of social approbation bias in self-rating scales (Greenberg and Weiss, 2012), we controlled for the variables using a hierarchical multiple regression when analyzing the relationship between cultures and self-construal. The skewness coefficients of the self-construal dimensions were between −0.52 and 2.46, and the kurtosis coefficients were between 0.37 and 3.98. A skewness coefficient lower than 3 and a kurtosis coefficient lower than 8 indicates an approximately normal distribution (Kline, 2010).

The significance of the homogeneity of variance test for each dimension of self-construal was greater than 0.05; the value of the Durbin-Watson test for each dimension of self-construal was in the range of 1.52–1.88, and the residuals were independent. The correlation coefficients between the independent variables were all below 0.5, and the tolerance level exceeded 0.95. The variance expansion factor fell in the range of 1.01–1.03. Thus, there was no multicollinearity problem and the conditions for regression analysis were met. A regression model was formed under the three cultural identities, using gender, growing up environment, and social desirability as the first level of entry in the multilevel regression as the baseline model. Each of the three cultural identities was then entered into the second level of the regression process, as shown in Tables 2, 3.

**Table 2:** A multilayer regression model on the relationship between cultural identities and self-construal (*n* = 460).

Individuality	*B*	SE	*t*	*p*	*B*	SE	*t*	*p*	*B*	SE	*t*	*p*
Gender	−0.84	0.303	−2.77	0.006	−0.65	0.311	−2.09	0.037	−0.652	0.312	−2.09	0.037
Growing up environment	0.952	0.308	3.067	0.002	1.11	0.321	3.469	0.001	1.092	0.318	3.434	0.001
Social desirability	−0.06	0.029	−2.14	0.033	−0.08	0.031	−2.62	0.009	−0.078	0.031	−2.52	0.012
ICI	0.162	0.029	5.681	<0.001								
CCI					0.009	0.022	0.403	0.687				
NCI									−0.004	0.027	−0.160	0.873
R^2^		0.11				0.043				0.037		
**Δ**R^2^		0.06				<0.001				<0.001		
F for**Δ**R^2^		32.27^***^				0.16				0.26		
Relationality	*B*	SE	*t*	*p*	*B*	SE	*t*	*p*	*B*	SE	*t*	*p*
Gender	−0.01	0.335	−0.035	0.972	−0.011	0.319	−0.035	0.972	0.185	0.321	0.575	0.566
Growing up environment	−0.96	0.341	−2.82	0.005	−0.627	0.329	−1.91	0.057	−0.835	0.327	−2.55	0.011
Social desirability	0.198	0.033	6.069	<0.001	0.166	0.031	5.295	<0.001	0.161	0.032	5.080	<0.001
ICI	0.026	0.032	0.823	0.411								
CCI					0.147	0.022	6.571	<0.001				
NCI									0.174	0.028	6.198	<0.001
R^2^		0.076				0.154				0.146		
**Δ**R^2^		0.001				0.08				0.07		
F for**Δ**R^2^		0.68				43.18^***^				38.41^***^		
Collectivity	*B*	SE	*t*	*p*	*B*	SE	*t*	*p*	*B*	SE	*t*	*p*
Gender	1.20	0.363	3.319	0.001	1.203	0.333	3.614	<0.001	1.549	0.319	4.851	<0.001
Growing up environment	−0.93	0.369	−2.52	0.012	−0.455	0.343	−1.33	0.186	−0.707	0.325	−2.18	0.030
Social desirability	0.103	0.035	2.929	0.004	0.058	0.033	1.780	0.076	0.037	0.031	1.173	0.241
ICI	0.036	0.034	1.052	0.293								
CCI					0.209	0.023	8.918	<0.001				
NCI									0.318	0.028	11.40	<0.001
R^2^		0.052				0.191				0.261		
**Δ**R^2^		0.002				0.141				0.211		
F for**Δ**R^2^		1.58				79.53^***^				130.5^***^		

^*^p < 0.05, ^**^p < 0.01, ^***^p < 0.001; ICI, Individualistic Cultural Identity; CCI, Confucian Cultural Identity; NCI, National Cultural Identity.

**Table 3:** A multilayer regression model on the cultural identities and self-construal (n = 460).

Autonomy	*B*	SE	*t*	*p*	*B*	SE	*t*	*p*	*B*	SE	*t*	*p*
Gender	−0.45	0.286	−1.58	0.115	−0.403	0.284	−1.42	0.157	−0.321	0.284	−1.13	0.259
Growing up environment	0.458	0.292	1.569	0.117	0.603	0.293	2.058	0.040	0.550	0.289	1.901	0.058
Social desirability	0.108	0.028	3.883	<0.001	0.094	0.028	3.365	0.001	0.088	0.028	3.155	0.002
ICI	0.050	0.027	1.857	0.064								
CCI					0.047	0.020	2.336	0.020				
NCI									0.076	0.025	3.068	0.002
R^2^		0.048				0.052				0.060		
**Δ**R^2^		0.009				0.010				0.019		
F for**Δ**R^2^		3.45				5.45^*^				9.41^***^		
Equality	*B*	SE	*t*	*p*	*B*	SE	*t*	*p*	*B*	SE	*t*	*p*
Gender	−0.003	0.227	−0.015	0.988	0.028	0.232	0.121	0.904	0.089	0.227	0.393	0.694
Growing up environment	−0.120	0.234	−0.510	0.610	−0.22	0.236	−0.951	0.342	−0.189	0.231	−0.819	0.413
Social desirability	−0.012	0.022	−0.552	0.581	−0.01	0.023	−0.119	0.905	−0.018	0.022	−0.799	0.425
ICI	0.057	0.016	3.554	<0.001								
CCI					0.017	0.022	0.779	0.436				
NCI									0.085	0.020	4.293	<0.001
R^2^		0.029				0.004				0.041		
**Δ**R^2^		0.027				0.001				0.038		
F for**Δ**R^2^		12.63^***^				0.61				18.43^***^		

^*^p < 0.05, ^**^p < 0.01, ^***^p < 0.001; ICI, Individualistic Cultural Identity; CCI, Confucian Cultural Identity; NCI, National Cultural Identity.

Tables 2, 3 show that controlling for gender, growing up environment, and social desirability, individualistic cultural identity significantly and positively predicted individuality, equality, and borderline significantly and positively predicted autonomy. Confucian cultural identity significantly positively predicted relationality, collectivity, and autonomy. National cultural identity significantly positively predicted relationality, collectivity, autonomy, and equality.

### 2.4 Discussion

The links between the three cultural identities and the components of self-construal have overlapping and centrally distinct parts, reflected in the coefficients of determination of the regression analyses. These findings suggest that the cultural identities are not in complete opposition to each other, but share some common elements. As some scholars have argued, when an individual acquires knowledge and customs from a new culture, the remembered ancestral inheritance of the knowledge and customs is not replaced, but rather retained. In addition, any given individual may have multiple cultural identities that do not have conflicting psychological consequences (Chiu and Hong, 2009).

## 3 Study 2: measurement using the cultural icon priming paradigm

### 3.1 Objective

To reveal the mechanisms of the relationship between culture and self-construal by having participants view and interpret cultural icons to activate culture (individualistic, Confucian or national).

### 3.2 Experimental design

The study used a one-way four-level between-subjects design. The independent variable was icon priming, which consisted of four levels: control group icon priming, individualistic culture icon priming, Confucian culture icon priming, and national culture icon priming. The dependent variables were the corresponding self-construal dimensions.

### 3.3 Methods

#### 3.3.1 Preparation of priming materials

We extensively examined everyday people, folklore, architecture, food, books, literature, movies, and sports, and consulted a team of 7 psychology researchers to find suitable icons to represent individualistic, Confucian, and national cultures. Sixty representative cultural icons were initially selected, and 54 participants [30 females; 24 males, mean age 26.67 years (*SD* = 5.45)] assessed the images. The cultural representativeness index for each icon ranged from 1 to 6, with higher scores indicating greater representativeness. Referring to existing cultural icon priming studies (Liu et al., 2017; Hu et al., 2018), the top 9 icons from each culture, with representativeness scores greater than 4.3, were selected as the priming icons.

The individualistic cultural symbols were, in descending order of representativeness: Jesus, Santa Claus, the Statue of Liberty, Western cutlery, hamburgers, the Eiffel Tower, Hollywood, Superman, and surfing. The selected Confucian cultural icons were: Confucius, Analects of Confucius, Zhu Xi’s character for filial piety (孝), the Chinese character for benevolence (仁), the performing filial piety picture (行孝图), Mencius, the Chinese characters for “Honoring Ancestors” (光宗耀祖), Tomb Sweeping Day, and the Chinese characters for “brotherhood” (手足情深). The selected national cultural icons were Lei Feng (雷锋), Serve the People (为人民服务), the National flag of the People’s Republic of China, Chinese dream, Monument to the People’s Heroes, Tiananmen, the Chinese women’s volleyball team being honored, China’s space exploration, and the movie Wolf Warrior II. The representativeness scores for the main icons of the three cultures are shown in Table 4.

**Table 4:** Ranking of the representativeness of the cultural icons of the three cultures.

Individualistic culture	*M*(*SD*)	Confucian culture	*M*(*SD*)	National culture	*M*(*SD*)
Jesus	5.67(0.51)	Confucius	5.72(0.52)	Lei Feng	5.55(0.73)
Santa Claus	5.40(0.82)	Analects of Confucius	5.57(0.73)	Serve the People	5.50(0.90)
Statue of Liberty	5.16(0.89)	The Chinese characters “filial piety”(孝)	5.16(0.97)	Nationalistic flag of the People’s Republic of China	5.47(0.88)
Western cutlery	5.05(0.89)	The Chinese characters “benevolence”(仁)	5.09(0.91)	the Chinese Dream	5.41(0.78)
Hamburgers	4.86(1.19)	The performing filial piety picture(行孝图)	5.07(0.81)	Monument to the People’s Heroes	5.14(0.93)
Eiffel Tower	4.86(0.98)	Mencius	5.00(1.10)	Tiananmen	5.14(1.07)
Hollywood	4.82(1.08)	The Chinese characters “Honoring Ancestors” (光宗耀祖)	4.68(1.03)	Chinese women’s volleyball team being honored	5.05(0.89)
Superman	4.73(1.02)	Tomb Sweeping Day	4.45(1.04)	China’s space exploration	4.41(1.42)
Surfing	4.64(1.20)	The Chinese characters “brotherhood” (手足情深)	4.45(1.21)	The Movie Wolf Warriors II(战狼2)	4.32(1.47)

#### 3.3.2 Participants

Standard requirements of the Biomedical Research Ethics Committee of Nanjing Normal University were followed. G*Power software was used to estimate the sample size. Under the conditions of one-way ANOVA with an effect size of 0.3, an alpha error of 0.05, statistical power (1-β) of 0.8, and the 4 subgroups, a sample of 128 participants was needed. In this study, 170 participants were recruited, of which 5 failed to understand the instructions accurately and thus, their data were not entered for statistical processing. The final number of participants was 165, of which 76 (46%) were female and 89 (54%) were male, with a mean age of 21.82 years (*SD* = 1.62).

#### 3.3.3 Tools

The PSCS (Li and Wang, 2023) was used. Cronbach’s α coefficients for the subscales in this study ranged from 0.67–0.88.

#### 3.3.4 Procedures

After informing the participants about the procedure, an informed consent agreement was reached. Cultural priming followed the approach of previous studies (Liu et al., 2017) with participants divided into four groups using randomization and the equidistant sampling method. The experimental procedure was divided into two stages: priming and measurement. During the priming stage, the instructions for the experiment were given. Participants were advised that a series of pictures would be displayed in the center of the computer screen. Each picture would be displayed for 12 s, with a total duration of 2 min for all the images. The series concluded with a summary table of all the pictures seen. After the final image, participants were asked to write approximately 100 words on paper about their experience of viewing the images, taking no more than 10 min. Then, the control group was primed with natural landscape pictures, and experimental group 1 was primed with individualistic culture icons. Experimental group 2 was primed with Confucian cultural icons, and experimental group 3 was primed with national culture icons. Participants checked items from a list based on the materials viewed for the priming of the manipulated icons, which were judged according to the relevance of what was written for the priming content (Hu et al., 2018). The materials were analyzed afterwards and participants’ writing was closely examined in relation to the priming icons. The priming stage was followed by an assessment stage in which participants were asked to complete the PSSC. Participants were paid for their time on completion of the experiment.

### 3.4 Results

#### 3.4.1 Basic scores for the priming experiments

Table 5 shows results of the priming experiment, comparing the scores of the control participants and each experimental group.

**Table 5:** Basic scores for the priming experiments (*n* = 165).

	Control Group (*n* = 42) *M*(*SD*)	Group 1(*n* = 40) *M*(*SD*)	Group 2(*n* = 43) *M*(*SD*)	Group 3(*n* = 40) *M*(*SD*)
Individuality Relationality Collectivity Autonomy Equality	3.98(0.71) 4.75(0.62) 4.21(1.05) 4.64(0.61) 4.71(0.86)	4.38(0.65) 4.71(0.69) 4.11(0.77) 4.72(0.73) 4.96(0.62)	4.19(0.77) 5.12(0.74) 4.31(0.64) 4.71(0.77) 4.57(0.98)	4.01 (0.67) 4.86(0.79) 4.61(0.80) 4.97(0.72) 5.01(0.74)

#### 3.4.2 Results of multivariate ANOVA between experimental and control groups

First, a multifactor ANOVA was used to analyze whether there was a significant interaction between culture types and growing up environment and gender. No significant interaction was found (significance levels in the range 0.229–0.972). Table 6 lists the results of the ANOVA of the polycultural self-construal on the four levels of the independent variable. Significant differences were found between the groups on the individuality dimension. *Post hoc* tests showed a highly significant difference between the control group and the individualistic culture priming group, with a significance level of *p* = 0.01 [95% confidence interval (CI) 0.099, 0.712], and between the individualistic and national culture priming group, a significance level of *p* = 0.022 [95% CI 0.065, 0.685]. These results suggest that for individuality, the individualistic culture priming group was significantly higher than the control and national culture priming groups.

**Table 6:** One-way ANOVA results.

	*df*	Mean square	*F*	*p*	η_p_^2^
Individuality	3	1.421	2.88	0.038	0.051
Relationality	3	1.400	2.77	0.044	0.049
Collectivity	3	1.918	2.79	0.042	0.049
Autonomy	3	1.139	2.38	0.097	0.038
Equality	3	1.825	2.74	0.045	0.048

Significant differences were found between the groups on the relationality dimension. *Post hoc* tests showed a significant difference between the control group and the Confucian culture priming group, with a significance level of *p* = 0.019 [95% CI −0.671, −0.061]; and between the Confucian and the individualistic culture priming group, a significance level of *p* = 0.011 [95% CI 0.095, 0.712]. The Confucian culture priming group was significantly higher than the control group and the individualistic culture priming group, increasing the relationality of the self-construal.

Significant differences were found between the groups on the collectivity dimension. *Post hoc* tests showed a significant difference between the control group and the national culture priming group, with a significance level of *p* = 0.029 [95% CI −0.766, −0.042]. Meanwhile, there was a highly significant difference between the individualistic culture priming group and the national culture priming group, with a significance level of *p* = 0.01 [95% CI −0.872, −0.140]. These suggest that for individuality, the priming effect of national culture is significantly higher than that of the control and individualistic culture priming groups.

There were borderline significant differences between the control and experimental groups on the autonomy dimension. *Post hoc* tests revealed a significant difference between the national culture priming group and the control group with a significance level of *p* = 0.013 [95% CI 0.085, 0.722].

There were significant differences between the groups in the equality dimension of self-construal. *Post hoc* tests showed a significant difference between the individualistic culture priming group and the Confucian culture priming group with a significance level of *p* = 0.03 [95% CI 0.039, 0.747]. There was a significant difference between the Confucian and national culture priming group with a significance level of *p* = 0.015 [95% CI −0.797, −0.089].

### 3.5 Discussion

The cultural icon priming experiment explored the mechanism between culture and self-construal from the perspective of polyculturalism. The results indicated that (1) compared with the control and experimental groups, the individualistic culture priming brought about a significant increase in individuality; the Confucian culture priming generated a significant increase in relationality, and national culture priming significantly increased collectivity and autonomy; and (2) comparing experimental groups, the individualistic culture priming brought about a more significant increase in individuality than the national culture priming; Confucian culture priming can bring about a more significant increase in relationality than individualistic culture priming; the national culture priming can create a more significant increase in collectivity than individualistic culture priming; individualistic culture priming can bring about a more significant increase in equality than the Confucian culture priming; and national culture priming can generate a more significant increase in equality than the Confucian culture priming. There were no significant differences between the three cultural priming types in terms of autonomy.

In summary, self-construal has a polycultural nature, and individualistic, Confucian, and national cultures have both idiosyncratic and overlapping parts in relation to the pluralistic connotations of the self-construal.

## 4 Study 3: content analysis study using the cultural icon priming paradigm

### 4.1 Objective

This part of the study combined the culture-initiated experimental paradigm and the content analysis method. Using different methods to study the same topic can increase the credibility of findings. Kuhn and McPartland (1954) created the Twenty Statements Test in their study of self-concepts. The logic behind Ten Statements Test (TST) is that humans are self-evident at the conscious level of mind and behavior, and that an individual’s introspective report of “who I am” reflects their true self-concept. The TST is an important method in self-construal research (Triandis et al., 1990; Hong et al., 2001; Spencer-Rodgers et al., 2009; Thomas et al., 2019).

### 4.2 Experimental design

The study used a one-way four-level between-subjects design. The independent variable was cultural icon priming, including four levels: control group, individualistic, Confucian, and national culture icon priming group; and the dependent variables were the corresponding self-construal dimensions.

### 4.3 Methods

#### 4.3.1 Participants

Again, the study met requirements of the Biomedical Research Ethics Committee of Nanjing Normal University. G*Power software was used to estimate the sample size. A sample size of 128 participants was needed under the conditions of one-way ANOVA with an effect size of 0.3, an alpha error of 0.05, statistical power (1-β) of 0.8, and 4 subgroups. In this study, 184 participants were recruited. Six participants’ data were not entered for statistical processing because they failed to understand the instructions. The final number of participants was 178, of which 91 (51.12%) were female and 87 (48.88%) were male, with a mean age of 22.2 years (*SD* = 5.45).

#### 4.3.2 Tools

The cultural icon priming material is shown in Table 4. The TST was used. Although early studies required participants to give 20 different responses to the question of “Who am I?,” Bochner (1994) found that 20-item responses were too long and that many participants dropped out of the free-form test after 10 responses. Hong et al. (2001), in their cultural icon priming study, also asserted that 20-item responses increased participant fatigue, and therefore, used ten statements. Based on these findings, this study asked participants to complete 10 “I am …” sentences.

#### 4.3.3 Procedures

Participants were informed of the basic procedure, and gave their informed consent to take part. They were divided into four groups through the principle of randomization using the equidistant sampling method. The experimental process was divided into two stages, a priming and a measurement stage, and the same procedures were followed as for Study 2.

#### 4.3.4 Coding schemes

Each participant’s “I am …” narratives were qualitatively processed using content analysis based on Kuhn and McPartland (1954). Each simple sentence was used as the smallest unit of analysis. Complex sentences were coded according to their meaning decomposition, and if the sentence was so complex that it was difficult to break down—as in the case of “I am a rational yet contradictory complex,” it was identified as related to self-reflection. Table 7 shows the coding scheme for this study based on the conceptual definitions of the dimensions of the polycultural self-construal and with reference to the coding schemes of related studies (Chen, 1994; Zheng and Huang, 1997).

**Table 7:** Coding schemes.

Categories	Examples
Individuality: Physiological characteristics Hobbies Uniqueness Self-interest Personal experience	I am long-haired; I am big-eyed. I am a person who likes take walks; I’m a person who likes to sing with my friends. I am unique. I am someone who needs to be cared for. I am someone who received a first-class scholarship.
Relationality: Family roles Friend character Lovers’ role Intimate relationship expression	I am a mother (wife, father, son, etc.). I am his best friend. I am the one who loves my girlfriend. I am the person who loves her; I am the person who wants my parents to be healthy.
Collectivity: age gender Social identity Professional Roles	I am Generation Z. I am a person. I am a man; I am a woman. I am Chinese; I am a Party member; I am a soldier; I am a teacher.
Autonomy: agency initiative transcendence	I am an autonomous person; I am a lazy person. I am a striver; I am a procrastinator. I am a transcendent; I am a daredevil.
Equality: Emphasis on equal rights Expression of the relationship between superiors and subordinates Emphasis on hierarchy	I am an egalitarian; I am a lover of justice. I am a subordinate; I am a superior. I am an opponent of patriarchy.
Other categories: Self-criticism Abstract statements Complex statements	I am a person who needs to reflect on life. I am grass, I am now, I am poison. I am a rotten person who cries, laughs and makes a mess.

Six dimensions were used to evaluate participants’ response information, namely individuality, relationality, collectivity, autonomy, equality, and others. Individuality involved individual-oriented statements, including the individual’s physical characteristics, interests, uniqueness, boundedness, self-interests, and experiences. Relationality included intimacy-oriented statements, mainly reflecting the individual’s roles in the family, and as a friend or lover. Collectivity involved group-oriented statements, including regarding gender, age, ethnicity, nationality, place of origin, social identity and social occupation. Autonomy incorporated statements about the self-governance of social life that relate to the characteristics of the individual’s initiative, agency, and willpower to challenge difficulties. Equality included statements reflecting the equality or inequality of an individual’s relationship with others or groups. The final ‘other’ category included statements that could not be clearly judged as belonging to one of the other five categories, and which were closely related to self-criticism.

### 4.4 Results

#### 4.4.1 Reliability analysis of raters

To ensure the objectivity of the coded results, references were made to the TST reliability determination index from relevant studies. There are four common methods for assessing the consistency of TST raters: (1) The rater consultation assessment method where raters discuss inconsistent items after coding individually, until agreement is reached (West et al., 2018). (2) The percentage of consistency method where a portion of the sample from the results completed by different coders is reviewed, and the consistency agreement percentage calculated (Grace and Cramer, 2003; Gatersleben et al., 2017). (3) Calculation of internal consistency alpha coefficients, as used by Spencer-Rodgers et al. (2009). However, whether alpha coefficients can be used for multidimensional scales is controversial (Reis and Judd, 2011) and (4) Calculation of kappa coefficients, currently the most commonly used method for assessing consistency of categorical data. This study combined methods 2 and 4. The author completed all coding based on the coding scheme, and then recruited a PhD student for additional coding. The student was familiarized with the coding scheme without being informed of the purpose of the study. He was given data for practice purposes, and parts of the exercise were clarified through that process. In the end, he coded 64.04% of the total material using the coding scheme. The authors computed this portion of the co-coded material and found 78% agreement between the two raters and a Cohen’s kappa coefficient of 0.674 (*p* < 0.001). Kappa values between 0.40 and 0.60 are acceptable according to Fleiss (1981), and thus, the objectivity of the data can be assured to some extent.

#### 4.4.2 Analysis of the proportions of responses on each dimension of self-construal

The importance of theoretical dimensions and tests of dimensional relationships can be obtained by analyzing the proportion of participants’ responses to items on each dimension (Watkins et al., 1997). Response proportions were calculated as the proportion of statements that responds to the same dimension out of the total number of statements, and then the size of the proportions for each dimension was compared. From this it was possible to characterize the structure of the self (Spencer-Rodgers et al., 2009). Table 8 shows the proportion of responses on each dimension of the polycultural self-construal for the four experimental groups.

**Table 8:** Proportion of responses on each dimension of self-construal in TST (*n* = 178).

	Control group (*n* = 43)		Group 1 (*n* = 47)		Group 2 (*n* = 43)		Group 3 (*n* = 45)	
	Frequency	%	Frequency	%	Frequency	%	Frequency	%
Individuality	141(1.5)	32.6	165(2.9)	34.9	114(−1.8)	26.3	113(−2.6)	24.9
Relationality	108(−0.8)	25	110(−1.8)	23.3	135(2.6)	31.2	120(0.7)	26.4
Collectivity	94(−0.9)	21.8	95(−1.9)	20.1	102(0.2)	23.6	126(2.6)	27.8
Autonomy	24(−0.8)	5.6	30(0.0)	6.3	28(0.1)	6.5	32(0.7)	7.0
Equality	19(−1.4)	4.4	32(1.1)	6.8	19(−1.4)	4.4	33(1.6)	7.3
Others	46(1.9)	10.6	41(0.2)	8.7	35(−0.3)	8.1	30(−1.7)	6.6
Sum	432	100	473	100	433	100	454	100

The two-way categorical data qualified for the chi-square test (R × C). Statistical tests indicated that the differences in polycultural self-construal scores under the four experimental treatments were statistically significant (χ^2^ = 27.45, *p* = 0.025). Between group differences were judged based on adjusted standardized residuals. Differences between observed and expected frequencies were considered statistically significant when the absolute value of the adjusted standardized residuals (AVASR) was greater than 2 (Agresti, 2002, p. 38). Table 8 shows that the AVASR of individuality in the individualistic culture priming group was 2.9, which indicates that the individualistic culture priming improved the level of individuality relative to the control group. Between the experimental groups, the AVASR of the difference between the individualistic culture priming and the Confucian culture priming reached 4.7, and the AVASR of the difference with the national culture priming reached 5.5, indicating that the effect of the individualistic culture priming was greater than that of the Confucian and national culture priming. In terms of collectivity, the AVASR of the national culture priming group was 2.6, while the AVASR of individuality was 1.9, which indicates that the priming of national culture increased the level of collectivity and decreased the level of individuality. For autonomy, the priming of cultural icons did not cause significant changes and there were no significant differences between the experimental groups. In terms of equality, the AVASR of the difference between the individualistic culture priming and the control group was 2.5, and the AVASR of the difference between the national culture priming group and the control group was 3, indicating that the individualistic and national culture priming increased the level of equality.

### 4.5 Discussion

The relationship between culture and self-construal was explored again in Study 3. When the control and cultural priming groups were compared, the individualistic culture priming significantly increased the self-construal of individuality and equality; the Confucian culture priming significantly increased relationality, and national culture priming significantly increased collectivity and equality. When the cultural priming groups were compared in terms of individuality, the individualistic culture priming was significantly higher than the Confucian and national culture priming. In terms of relationality, the Confucian culture priming was significantly higher than the individualistic culture priming. For collectivity, the national culture priming was significantly higher than the individualistic and Confucian culture priming. In terms of autonomy, no significant difference was found among the three priming types. For equality, the national and individualistic culture priming were significantly greater than the Confucian culture priming. These results were generally consistent with those of Study 2.

## 5 General discussion

The three studies explored the mechanisms of culture and self-construal in multiple ways. Cultural identity research explores the relationship between culture and self-construal at a conscious level, while cultural icon initiation involves the subconscious level. Cultural identity reflects the role of the subject, and cultural icon activation reflects the role of the cultural object. Combining the two forms reveals the connotation of the relationship between culture and self-construal in a more complete way.

In the measurement study of cultural identity, individualistic cultural identity was not significantly correlated with national cultural identity, whereas in the later experimental study the two cultures were somewhat correlated in terms of equality. Possible reasons for the inconsistencies are that cultural identity was measured by self-report, where participants had a higher level of awareness, whereas participation in the priming experiment was more implicit in nature. Different levels of cognitive awareness may have contributed to the differences.

Differences in the response of different components of self-construal to cultural priming can be explained in depth at motivational and cognitive levels. “Unlike trait-oriented research, motivational research articulates the mechanisms by virtue of which personality content is converted into specific behaviors across contexts and time, and utilizes an interactional perspective to consider person-situation relationships” (Guo and Hu, 2015, p. 1491). This perspective implies that components of the self-construal are differently linked to the motivational system of excitation at the time of cultural icon priming in terms of the strength of their connection, and thus, perform differently. In addition, the differences in relevant priming effects may be related to self-relevant cognitive processing styles. Researchers have found that self-construal is characterized by some stability and some activation by the environment (Wu, 2018). Different components of self-construal respond variably to contextual cues. One mechanism may be that different components of the self rely differently on episodic and semantic self-knowledge (Wang et al., 2016).

Study 3 differed from Study 2 in two ways. First, the priming effect of individualistic culture on individuality was found to be significantly higher in Study 3 than that of Confucian culture on individuality, whereas there was no significant difference between the two in Study 2. Second, in terms of autonomy, there was no significant difference in the priming effect of national culture in Study 3 compared with the control group, whereas there was a significant difference in Study 2. These differences may reflect differences in the way the two self-construals were assessed. The TST paradigm judges the connotation of self-concept primarily based on response proportions, whereas the PSCS is based on response strengths. The combination of response proportions and strengths is complementary and enhances the reliability of the findings. However, the difference in methods may have brought about some of the discrepancies in the findings.

In summary, the self-construal of Chinese people in the era of globalization has a polycultural connotation, making it difficult to understand the self-construal phenomenon through a specific culture. As some researchers have noted, “Under the vision of polyculturalism, the management of multicultural identities by individuals is not a simple either/or choice, not a process of giving up original cultural identities in order to obtain mainstream group identities, but a process of coexistence and mutual support among different identities” (Wu et al., 2017, p. 180).

## 6 Conclusion

There are three main types of culture in human society, namely, individual-oriented, relationship-oriented and social-oriented cultures. The three studies in this paper found that, in the era of globalization, individuals’ self-construal is polycultural. Individual-oriented culture mainly affects the individuality and equality of self-construal. Relational-oriented culture mainly influences the relationality of self-construal, and social-oriented culture mainly affects the collectivity and equality of self-construal. There was no significant difference in the effects of the three cultures on autonomy. The three cultures have some specific links to particular aspects of self-construal but also share in the process of self-construal more generally.

## Data availability statement

The original contributions presented in the study are included in the article/supplementary material, further inquiries can be directed to the corresponding author.

## Ethics statement

The studies involving humans were approved by the Biomedical Research Ethics Committee of Nanjing Normal University. The studies were conducted in accordance with the local legislation and institutional requirements. The participants provided their written informed consent to participate in this study.

## Author contributions

KL: Conceptualization, Investigation, Methodology, Project administration, Resources, Writing – original draft, Writing – review & editing, Data curation, Formal analysis, Funding acquisition, Software, Supervision, Validation. FW: Conceptualization, Methodology, Supervision, Writing – review & editing, Data curation. ZP: Data curation, Methodology, Writing – review & editing.
